# Proteasome and Autophagy-Mediated Impairment of Late Long-Term Potentiation (l-LTP) after Traumatic Brain Injury in the Somatosensory Cortex of Mice

**DOI:** 10.3390/ijms20123048

**Published:** 2019-06-21

**Authors:** Lucia K. Feldmann, Florie Le Prieult, Vanessa Felzen, Serge C. Thal, Kristin Engelhard, Christian Behl, Thomas Mittmann

**Affiliations:** 1 Institute for Physiology, UMC of the Johannes Gutenberg University Mainz, Duesbergweg 6, 55128 Mainz, Germany; lucia.feldmann@charite.de (L.K.F.); florie.leprieult@gmail.com (F.L.P.); 2 Institute for Pathobiochemistry, UMC of the Johannes Gutenberg University Mainz, Duesbergweg 6, 55128 Mainz, Germany; Vanessa.Felzen@gmail.com (V.F.); cbehl@uni-mainz.de (C.B.); 3 Clinics for Anaesthesiology, UMC of the Johannes Gutenberg University Mainz, Langenbeckstraße 1, 55131 Mainz, Germany; thal@uni-mainz.de (S.C.T.); engelhk@uni-mainz.de (K.E.)

**Keywords:** traumatic brain injury, long-term potentiation, plasticity related proteins, proteasome, autophagy, somatosensory cortex

## Abstract

Traumatic brain injury (TBI) can lead to impaired cognition and memory consolidation. The acute phase (24–48 h) after TBI is often characterized by neural dysfunction in the vicinity of the lesion, but also in remote areas like the contralateral hemisphere. Protein homeostasis is crucial for synaptic long-term plasticity including the protein degradation systems, proteasome and autophagy. Still, little is known about the acute effects of TBI on synaptic long-term plasticity and protein degradation. Thus, we investigated TBI in a controlled cortical impact (CCI) model in the motor and somatosensory cortex of mice ex vivo-in vitro. Late long-term potentiation (l-LTP) was induced by theta-burst stimulation in acute brain slices after survival times of 1–2 days. Protein levels for the plasticity related protein calcium/calmodulin-dependent protein kinase II (CaMKII) was quantified by Western blots, and the protein degradation activity by enzymatical assays. We observed missing maintenance of l-LTP in the ipsilateral hemisphere, however not in the contralateral hemisphere after TBI. Protein levels of CaMKII were not changed but, interestingly, the protein degradation revealed bidirectional changes with a reduced proteasome activity and an increased autophagic flux in the ipsilateral hemisphere. Finally, LTP recordings in the presence of pharmacologically modified protein degradation systems also led to an impaired synaptic plasticity: bath-applied MG132, a proteasome inhibitor, or rapamycin, an activator of autophagy, both administered during theta burst stimulation, blocked the induction of LTP. These data indicate that alterations in protein degradation pathways likely contribute to cognitive deficits in the acute phase after TBI, which could be interesting for future approaches towards neuroprotective treatments early after traumatic brain injury.

## 1. Introduction

Traumatic brain injury (TBI) is one of the major global causes of morbidity and mortality in adults under 40 years of age [1,2]. Focal TBI leads to brain damage with variable lesion patterns including a necrotic lesion center surrounded by brain edema, vascular injury and diffuse axonal injury, all contributing to the heterogeneous clinical outcome [3,4]. Furthermore, focal TBI induces secondary brain damage during the first 48 h after TBI, mainly caused by inflammatory reactions, reactive gliosis as well as increased phagocytic activity and apoptosis [4,5,6,7,8]. An altered neurotransmitter and calcium release resulted in changes of neuronal network activity even in cells primarily unaffected by the TBI [9]. Furthermore, the distant contralateral brain hemisphere was shown to react to focal, ipsilateral impacts with regard to changes in gamma-aminobutyric acid (GABA) signaling and increased network excitability in mouse models [10,11] as well as in imaging studies in humans [12]. Post-traumatic, adaptive cellular processes affect the functional reorganization of the cortical networks as well as the neuronal loss resulting in either beneficial or detrimental effects with respect to the neurological outcome [13].

Reported symptoms after TBI include cognitive deficits, especially regarding consolidation of new information. Late long-term potentiation (l-LTP), as the persistent enhancement of synaptic transmission, is assumed to be the cellular correlate of learning and memory formation [14,15]. Expression of l-LTP is based on activity-induced calcium influx through N-methyl-d-aspartic acid (NMDA) receptors [16,17] and activation of protein synthesis processes [18]. Through modulation of postsynaptic protein homeostasis and architecture of the postsynaptic density [19], a selective strengthening of synapses and thus, memory formation, can be achieved, for example by phosphorylation of postsynaptic modulators, so called plasticity related proteins. It is well known that phosphorylation of the calcium/calmodulin-dependent protein kinase II (CaMKII) leads to the potentiation of existing receptors [20], activation of specific synapses [18,21] and strengthening of dendritic spines through F-actin polymerization [22]. Furthermore, the balance of two protein degradation systems, autophagy and proteasome activity, is crucial to achieve synaptic plasticity [23]. Decreased activation of the proteasome is related to impaired synaptic plasticity [23,24,25]. Moreover, the experimental activation of autophagy with mammalian target of rapamycin (mTOR) inhibitors such as rapamycin [26] as well as nanoparticles [27] suppresses LTP and influences neurotransmission [28]. Recent studies suggest that the differential activation of these two protein degradation systems is mediated by heat shock protein 70 (Hsp 70) [29,30]. Hsp 70 is activated through co-chaperones beclin2-associated anthanogenes (BAG). While BAG1 modulates Hsp 70 increasing proteasome activity, BAG3 controls BAG3-mediated selective macroautophagy [29,30,31]. Strikingly, shifts in BAG concentrations could be linked to pathological changes, e.g., in ageing [29,32]. In TBI, several synaptic properties appear to be affected leading to changes in receptor and transmitter concentrations as well as specific cell survival [9], as a consequence of dysregulation through excitotoxicity. Proteasome activation is impaired during the first week post-lesion [33] with increasing levels of proteolytic activity at later time points post-lesion [34]. Autophagy activity as indicated by LC3-II is increased reaching maximum levels three days post-TBI [35,36,37]. However, so far it is not known, how a focal TBI affects l-LTP in the ipsilateral and contralateral hemisphere of the somatosensory cortex at all, and if and how protein degradation systems as well as plasticity related proteins are involved in any potential changes of l-LTP during the acute, early phase (24–48 h) after the injury. Here, we present evidence for specific alterations in the protein homeostasis and in long-term synaptic plasticity already 24-48 h after TBI. Interestingly, the impaired l-LTP was observed exclusively in the ipsilateral hemisphere of the somatosensory cortex. Our results provide new information on the mechanisms underlying early cognitive impairments observed in patients after traumatic brain injury.

## 2. Results

### 2.1. Lesion Expansion in the Acute Phase after Controlled Cortical Impact (CCI)

**Figure 1:**
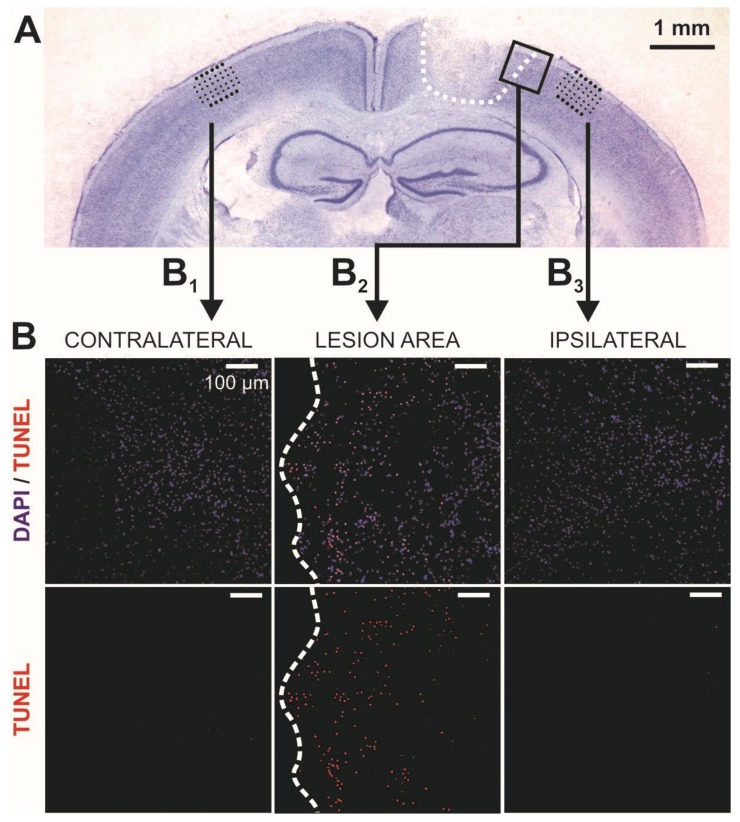
Histology of the controlled cortical impact (CCI) lesion. (**A**) Representative example of a Nissl stained coronal section of the sensorimotor cortex from a 20-day-old mouse at 1 day post-CCI. The black dotted squares represent the perforated microelectrode array (pMEA) chip position for monitoring extracellular signals, the recording electrodes were located in supra-granular layers 2/3. We recorded 1 mm lateral from the border of the lesion and in the homotopic region of the contralateral hemisphere and sham animals. (**B**) Some slices were treated with an in situ TUNEL assay to detect early signs of apoptosis. The resulting confocal images were taken in specific areas of the cortical slice as shown in Figure 1A (see arrows) and are shown at a higher magnification (the white scale bar corresponds to 100 µm). B1 shows an example of a confocal image of the homotopic area of the contralateral hemisphere, images in B2 were taken from the lesion area in the ipsilateral hemisphere, while images in B2 were derived at 1 mm distance from the border of the lesion in the ipsilateral hemisphere. Note that TUNEL positive cells were detected at the close border of the lesion (white dashed line). In contrast, at more distant areas ipsilateral (1 mm distance to the lesion) and contralateral, the MEA-recording sites revealed almost no signs of apoptosis.

In accordance with our previous study [11], the controlled cortical impact (CCI) induced a cortical lesion in the motor- (M1 and M2) as well as in somatosensory (S1) cortex of the mice. It measured 1.5–2 mm in mediolateral extent and extended along the rostrocaudal axis between 0 and −2 mm relative to bregma. The lesion size and location was highly reproducible and affected all cortical layers, as shown by the representative Nissl-stained coronal section of a CCI-treated brain (Figure 1A).

First, we evaluated an early TBI-induced potential spreading of cell death (apoptosis) in the areas of interest and performed in situ TUNEL labeling of DNA-strand breaks in all experimental groups. Apoptosis-positive cells could be identified mediolaterally up to a distance of 300–400 μm from the border of the lesion (Figure 1B_2_). Interestingly, the areas of our electrophysiological recordings in both, ipsi- and contralateral hemisphere revealed no significant expression of cell death in the early phase of TBI after 24 and 48 h (Figure 1B_1_,B_3_).

### 2.2. Impairment of Long-term Synaptic Plasticity 1 to 2 Days Post-Lesion

**Figure 2:**
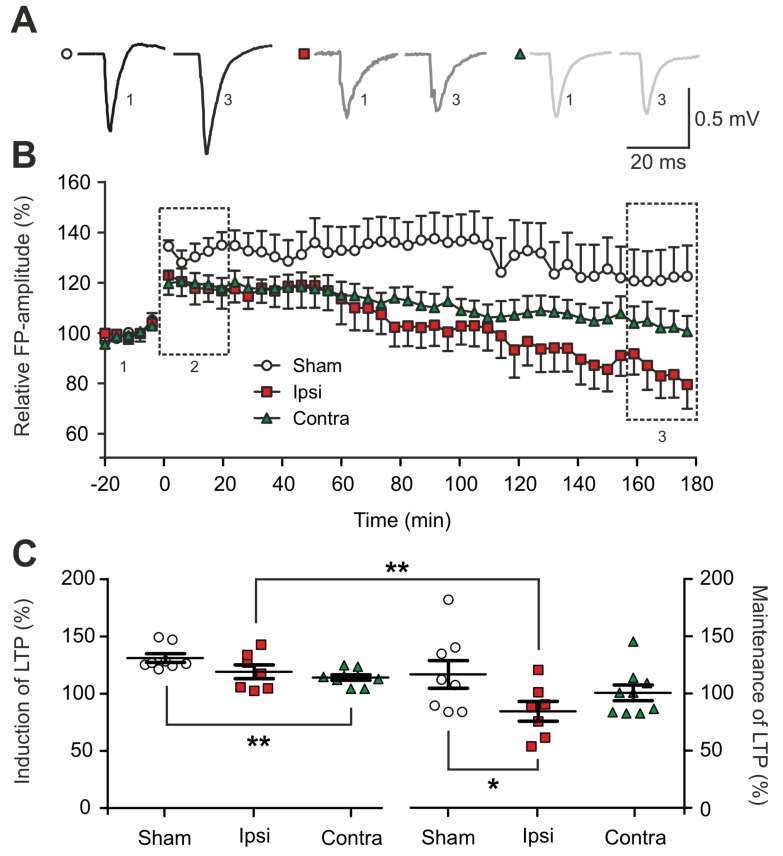
Impairment of long-term synaptic plasticity (late long-term potentiation (l-LTP)) in the early phase after traumatic brain injury (TBI). (**A**) Representative voltage traces of evoked extracellular field potentials (FPs) recorded before (Pre-TBS, baseline, 1 in (**B**)) and after electrical theta-burst stimulation (TBS) (post-TBS, maintenance, 3 in (**B**)) and under different experimental conditions at 1-2 days post-TBI (sham: *n* = 8 from 8 mice; ipsilateral: *n* = 7 from 7 mice; contralateral: *n* = 8 from 8 mice). (**B**) Time course of relative local field potentials (LFPs) amplitude pre- and post-TBS. After baseline recording (1), TBS protocol was applied and LFPs were recorded for 180 min; data points represent mean values of the electrophysiological recordings under different conditions (sham: *n* = 8 from 8 mice; ipsilateral: *n* = 7 from 7 mice; contralateral: *n* = 8 from 8 mice) relative to the recordings of the individual baseline, the error bars represent standard error of mean (SEM). The dashed rectangles represent the time windows considered for the induction (2: first 20 min of recording) and the maintenance of l-LTP (3: last 20 min of recording), as visualized in (**C**). The lesion significantly decayed the expression of ipsilateral l-LTP over the course of recording. (**C**) Scatter plots showing the induction (2 in (**B**)) and the maintenance (3 in (**B**)) of l-LTP in the different experimental conditions over a recording period of 20 min; data points represent the relative values of baseline stimulation acquired during the induction phase (**C** left) in the first 20 min post-TBS and the maintenance phase (**C** right) during the last 20 min; mean and SEM are indicated by the black horizontal lines. The asterisks indicate the significance of the tested data in one-way analysis of variance (ANOVA) and post hoc least significant difference (LSD), significantly different values are indicated as *: *p* < 0.05, **: *p* < 0.01.

Changes in initiation, regulation and maintenance of synaptic plasticity are crucial for functional reorganization processes in the post-traumatic brain. In this study, our focus was the expression and maintenance of l-LTP, since processes like protein synthesis and degradation might particularly influence l-LTP. Acute cortical slices were selected between bregma −0.5 and −1.5 mm in 1–2 days post-CCI animals. After positioning the 32-channel perforated microelectrode array (pMEA) chip in the supragranular layers and 1 mm away from the border of the lesion or in homotopic areas both contralaterally and in sham animals (Figure 1B), we recorded theta-burst stimulation (TBS)-induced l-LTP for a duration of 180 min (Figure 2A,B). As further described in the Methods section, TBS is an established repetitive high-frequency stimulation protocol for induction of synaptic plasticity.

Only measurements with a significant increase of FP-amplitudes in the first 20 min after TBS in all experimental conditions, when compared to the baseline signals, were included in the analysis. However, FP-amplitudes during induction of LTP were not significantly reduced in the ipsilateral hemisphere (119.3 ± 5.5%) (Figure 2C). With respect to the maintenance of l-LTPs (160 to 180 min post-TBS) we observed exclusively a significant reduction of the FP-amplitudes in the ipsilateral cortex when compared to sham-operated animals (sham: 116.9 ± 11.3%; ipsilateral: 84.6 ± 8.1%, *p* = 0.05; contralateral: 100.7 ± 7.3%) (Figure 2C). Furthermore, within one LTP experiment the ipsilateral cortex revealed a significant decay in the strength of l-LTP from the induction to its maintenance (*p* = 0.0066) (Figure 2C). Interestingly, the contralateral cortex revealed no changes in the maintenance of l-LTP in our recordings (Figure 2C).

### 2.3. Dysregulation of the Phosphorylation Level of Calcium-Calmoduline Dependent Kinase II α (CaMKIIα) 1 to 2 Days Post-Traumatic Brain Injury (TBI)

**Figure 3:**
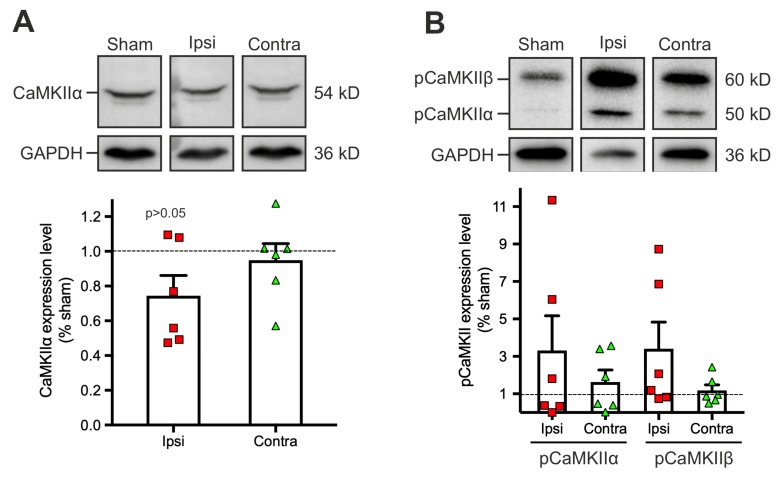
Phosphorylation levels of calcium-calmodulin dependent kinase II (CaMKIIα) in the acute period after TBI are not significantly altered. Exemplary Western blots of CaMKIIα (**A**) and phosphorylated CaMKII (pCaMKII) (**B**). In the lower section, protein levels from brain lysates of the respective cortical areas of interest are depicted in scatter plots for CaMKIIα (*n* = 6 from 6 mice for each experimental condition) and pCaMKII (*n* = 6 from 6 mice for each experimental conditions). Expression levels were quantified after normalization to the housekeeping protein GAPDH. The dashed line represents the CaMKIIα protein level in sham-operated animals, normalized to 1, the data points in the scatter plots represent the relative values for protein expression levels in the ipsilateral or contralateral hemisphere per animal, bars and error bars represent mean and SEM. For statistical analysis pairwise Mann-Whitney-U test was performed, they revealed no significant differences.

In search for potential cellular mechanisms underlying the observed changes in the strength of LTP induction and maintenance early after TBI, we investigated processes of protein homeostasis in the cortical tissue, initially focusing on changes in the level of the plasticity related protein calcium-calmoduline dependent kinase II α (CaMKIIα). Cortical tissue was collected from the same brain area where the electrophysiological recordings were performed. Quantification of the non-phosphorylated form of CaMKIIα did not disclose significant changes in neither of the hemispheres, although a tendency for a decreased CaMKIIα expression was observed ipsilaterally (sham: 1; ipsilateral: 0.7 ± 0.1, *p* = 0.16; contralateral: 0.9 ± 0.09) (Figure 3A).

When activated after synaptic stimulation like our tetanic LTP stimulation protocol, CaMKII is selectively phosphorylated on the amino-acid Thr286. Since during TBI the described calcium inflow could result in undirected phosphorylations, we examined the levels of CaMKII phosphorylation isoforms α and β (Figure 3B). In a sham condition, the basal phosphorylation levels of both isoforms (particularly pCaMKIIα) were low since no experimental l-LTP induction was performed before tissue collection. Albeit a trend to increase, basal phosphorylation levels of both pCaMKIIα (sham: 1; ipsilateral: 3.3 ± 1.7; contralateral: 1.6 ± 0.6) and pCaMKIIβ (sham: 1; ipsilateral: 3.4 ± 2.4; contralateral: 1.2 ± 0.9) were not significantly altered after the lesion (Figure 3B). Altogether, these results suggest that alterations of CaMKII expression and activation are not responsible for the observed ipsilateral impaired expression of l-LTP.

### 2.4. The TBI-Induced Impairment of Late Long-Term Potentiation (l-LTP) is Mediated by Altered Protein Degradation Systems

**Figure 4:**
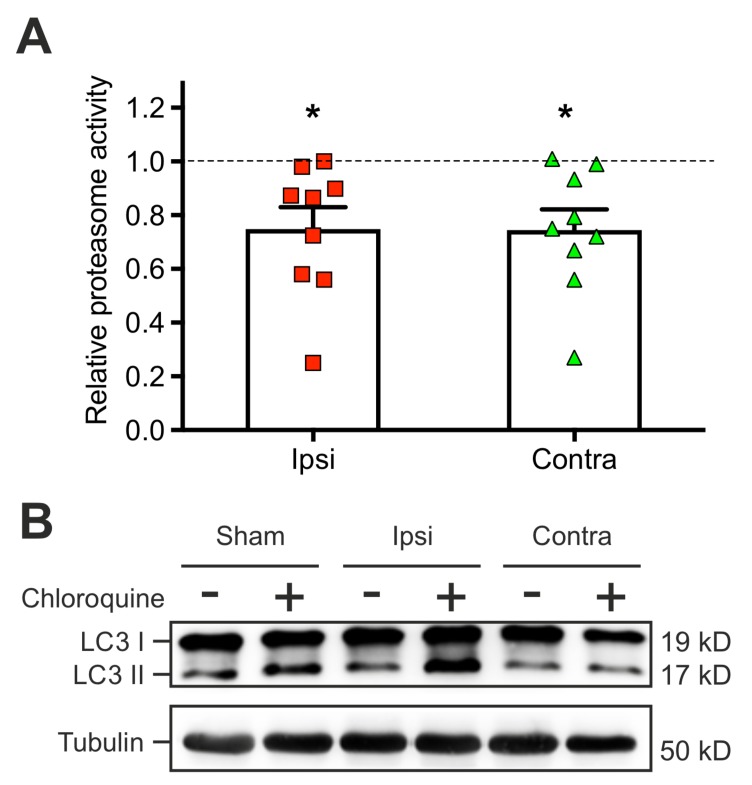
Altered protein degradation systems early after lesion. (**A**) Scatter plots representing the relative proteasome activity (9 mice for each experimental condition) measured in the ipsilateral area of electrophysiological recordings at 2 days post CCI. Data points represent the values for each animal per condition for protein expression levels in the ipsilateral or contralateral hemisphere relative to sham data. Mean and SEM are indicated by the bar plots and error bars. (**B**) Representative Immunoblot of the autophagosomal marker, light chain 3 (LC3) protein, from the cortical area of interest. Both cytosolic (LC3 I) and phosphatidylethanolamine conjugated forms of LC3 (LC3 II) were detected but only LC3 II was quantified in (**B**) after normalization to the housekeeping protein Tubulin. Note the impaired proteasome activity, while autophagy is increased compared to sham animals at the same time suggesting a shift in protein degradation systems. One-way ANOVA and D’Agostino and Pearson normality tests disclosed significant differences of proteasome activity in the ipsi- and contralateral hemisphere, levels of significance are indicated as *: *p* < 0.05.

Processes of l-LTP and the associated activation of the CaMKII have been tightly linked to protein homeostasis. Particularly, protein degradation plays an important role in synaptic plasticity by participating in both synapse formation and synapse elimination induced by neuronal activity [38]. Therefore, we investigated the status of two important protein degradation systems after lesion, the proteasome and the autophagy. The proteasome peptidase activity and the autophagic flux assays were investigated to quantify the activity of the respective pathways. Tissue samples were collected in each hemisphere in TBI-treated mice and in sham-operated animals at 2 days post-injury. The proteasome activity was measuered by an assay (see Methods). This revealed an impaired ubiquitin-dependent protein degradation in both hemispheres after TBI when compared to sham (ipsilateral: 0.75 ± 0.08, sham vs. ipsi: *p* = 0.02; contralateral: 0.74 ± 0.07, sham vs. contra: *p* = 0.019) (Figure 4A).

The second protein degradation system, the autophagic flux, was measured in acute brain slices of 3 mice for each experimental condition by detection of the LC3-II flux induced by bath application of rapamycin (Figure 4B). In contrast to the other protein degradation system (Figure 4A), the autophagic flux reacted differently to TBI: the CCI treated ipsilateral hemisphere showed an increase (3.2 ± 1.1, sham vs. ipsi: *p* = 0.037) in the autophagic flux compared to the sham condition, while the contralateral cortex showed a significant decrease in LC3-II flux (0.47 ± 0.3, sham vs. contra: *p* = 0.04).

**Figure 5:**
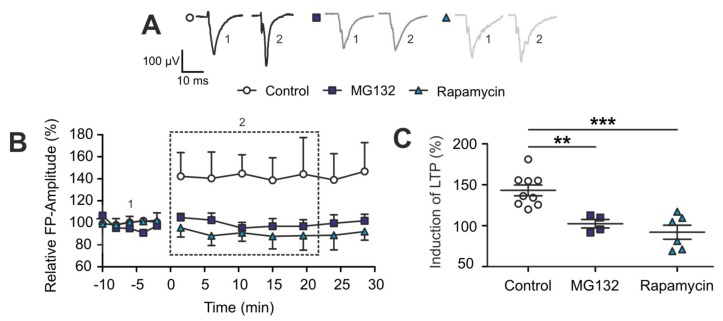
Pharmacological modulation of the two protein degradation systems induced TBI-like changes in l-LTP. (**A**) Representative voltage traces of evoked FPs recorded before (Pre-TBS, baseline, 1 in (**B**)) and after electrical TBS stimulation (post-TBS, induction, 2 in (**B**)) and under different pharmacological conditions. Cortical slices were used under control conditions (normal artificial cerebrospinal fluid (aCSF), control: *n* = 9 from 9 mice), in presence of MG132, a proteasome inhibitor (*n* = 4 from 4 mice), and in presence of rapamycin, an activator of autophagy (*n* = 6 from 6 mice). (**B**) Time course of relative FPs amplitudes pre- and post-TBS, data points represent the mean values for each condition (control: *n* = 9, MG132: *n* = 4, rapamycin: *n* = 6) relative to the individual recording’s baseline, error bars represent the SEM. The dashed rectangles represent the time windows considered for the induction phase of l-LTP (2: first 20 min of recording), as visualized in (**C**). Note, impairment of the proteasome or enhancement of autophagy suppressed the expression of l-LTP in wild type slices over the course of recording. (**C**) Scatter plot showing the abolished l-LTP under both pharmacological agents over a recording period of 20 min; data points represent the relative mean values for the first 20 min, the induction phase of LTP, for each recording; the mean and SEM are indicated by the black horizontal bars and error bars. The asterisks indicate the significance of the tested data in one-way ANOVA and post hoc LSD, significantly different values are indicated as **: *p* < 0.01, ***: *p* ≤ 0.001.

Next, we investigated the effects of the the detected TBI-induced alterations in the two protein degradation systems on synaptic plasticity. To do so, the strength of the proteasome and of the autophagy system were pharmacologically modified in brain sliced to mimic TBI situation in an otherwise untreated mouse brain by using either 10 µM MG132 for blocking the proteasome or 1 µM rapamycin for enhancing the autophagy. The expression of LTP was recorded in presence of one or the other pharmacological agent (Figure 5A,B).

During the first 30 min after induction of our LTP-stimulation protocol, no induction of LTP could be observed in neither of the two pharmacological conditions when compared to control slices in normal artificial cerebrospinal fluid (aCSF) (control: 143.2 ± 6.2%; MG132: 102.4 ± 4.5%, p_ctrl/MG132_ = 0.003; rapamycin: 92.0 ± 7.8%, p_ctrl/rapamycin_ = 0.0003) (Figure 5B,C). Since no potentiation occurred, the recordings were stopped at 30 min post-TBS. Taken together, these data suggest that the observed impairment of long-term synaptic plasticity (l-LTP) early after TBI in the ipsilateral hemisphere is strongly correlated with specific dynamic changes in the two protein degradation systems, the proteasome and autophagic flux.

## 3. Discussion

The acute phase following TBI is characterized in part by specific adaptive cellular processes within the surviving neuronal networks, and this is highly relevant for the final clinical outcome in TBI-patients, e.g., for cognitive and motoric rehabilitation [9]. Still, these mechanisms are not fully understood. We have recently shown that a focal cortical brain injury led to network hyperexcitability in the contralateral hemisphere as early as 24–48 h post-lesion and interestingly, it induced changes in GABAergic functions as well as in the subunit-composition of GABA-receptors on the m-RNA level [10,11]. Here, we focused on the balance of protein homeostasis, which is also crucial for neuronal function, especially for processes of synaptic plasticity: late-onset neural dysfunction following TBI was associated with changes in protein homeostasis and protein degradation, which might lead to the development of neurodegenerative diseases such as Parkinson’s disease and Alzheimer’s disease in humans [39,40,41,42,43] and in experimental mouse models [44]. Furthermore, even in healthy brains, synaptic plasticity is affected by a singular modification of one protein degradation pathway [23,24,25]. Interestingly, in this context a regulatory mechanism was identified contrarily linking both protein degradation systems, proteasome and autophagy, through heat-shock proteins [31]. This regulatory mechanism could be shown to be influenced by processes such as ageing [30,32], and could be similarly affected in pathological conditions such as trauma. Hence, the different modes of protein degradation could be interesting targets for early modification of acute TBI, as clinically approved medications, such as rapamycin interacting with autophagy, are already in clinical use for completely different pathologies.

### 3.1. Traumatic Brain Injury Induced an Early Impairment of Long-Term Synaptic Plasticity

We induced LTP in acute brain slices ex-vivo in-vitro with a TBS stimulation protocol, which is well established in our lab [45,46,47], and was modified in the present study to achieve stable potentiation for more than 120 min (l-LTP). One main result of our experiments is that during the early acute phase after TBI, maintenance of LTP in the ipsilateral hemisphere is significantly reduced in comparison to sham-operated animals. So far, research on TBI-induced changes in LTP led to controversial results. On the one hand, several electrical membrane properties mediating the action potential firing and the input resistance were reported to be changed [48], and the glutamatergic neurotransmission was increased [49]. On the other hand, hippocampal synaptic plasticity was reduced during the acute phase post lesion in rats [50] as well as at later time points [51,52]. One major parameter contributing to the different results is distance of the recording from the center of the injury [47]. Our results here complement these findings and indicate that synaptic plasticity at a distance of 1.5 mm from the border of the lesion in the ipsilateral hemisphere is impaired. Excessive neuronal hyperexcitability following focal brain injuries [48,49] may result in an imbalance of the transmitter systems that has been linked to impaired LTP before [8]. Furthermore, we showed in our TUNEL staining that although there was apoptosis found in the immediate vicinity of the lesion, there was no evidence for apoptosis in the adjacent recording areas, confirming that changes in electrophysiological measurements are unlikely to be a result of direct cellular damage, at least in the early phase after TBI.

In contrast, in the contralateral hemisphere no significant impairment of maintenance of LTP was observed while the induction of LTP was indeed, significantly reduced. Recent research, also from our lab, has investigated the importance of diaschisis in TBI [10,11] showing changed electrophysiological parameters in the contralateral hemisphere which are considered to contribute to recovery processes or, at least, maintain network stability in the ipsilateral hemisphere. In line with this hypothesis are the findings in the present study indicating that although initial potentiation is altered compared to sham, an overall maintenance of potentiation can be achieved as the undamaged contralateral hemisphere is not detrimentally affected by the lesion, but could rather reveal a compensatory function in this context.

### 3.2. Expression of Plasticity Related Proteins Is Not Significantly Altered Early after TBI

One major theory for maintenance of LTP is the “synaptic tagging and capture” hypothesis suggesting the importance of plasticity related proteins in the post-synapse for the maintenance of LTP [19]. After TBI, strikingly, the activation of regulatory proteins is often altered through phosphorylation which may be affected by post-traumatic increased calcium influx and thus, interfere with the function of protein signaling cascades. CaMKIIα is a major plasticity related protein involved in intracellular signaling pathways leading to differential synaptic strengthening [18,19,21], and it has been linked to proteasome activity as well [38] making it an interesting target protein for investigation of protein homeostasis. Our data suggest that the expression of CaMKIIα is not significantly altered post-TBI, although we observed a trend for reduced expression in the ipsilateral hemisphere. As we did not perform any stimulation protocols to induce LTP in the probes used for the Western blots, we expected a relatively low level of phosphorylation of CaMKII in sham animals. In contrast, we expected a TBI-induced intracellular calcium influx, which may last up to four days after CCI [53] and may potentially lead to a relatively higher level of phosphorylation in CCI animals, even without LTP induction. Interestingly, the detection of phosphorylated, thus activated, CaMKIIα was very variable. Although we observed a tendency for increased phosphorylation levels under conditions after TBI, especially in probes collected from the ipsilateral hemisphere, the unexpectedly high variability prevented any significant result of this experiment. However, our finding is in line with a study performed by use of a different TBI model, showing no upregulation of CaMKIIα phosphorylation in the ipsilateral hemisphere [54].

These data indicate that changes in long-term synaptic plasticity related to CaMKIIα activation are unlikely to be causally attributed to the impaired LTP observed in the electrophysiological measurements, but that a dysregulation after lesion, albeit non-significant, is in line with previous experiments [54]. Another plasticity related protein we tested, protein kinase Mξ (PKMξ), showed similar non-significant variable results (data not shown). It was excluded from this study as its significance for plasticity is controversially discussed [55,56,57,58]. Since activation of CaMKIIα is closely associated with protein degradation and related to synaptic plasticity as well [38], we further evaluated changes in protein homeostasis and investigated the other end of protein homeostasis, the protein degradation systems and their effects on synaptic plasticity.

### 3.3. TBI-Induced Early Bidirectional Changes in the Activity of Two Protein Degradation Systems

Here, we present evidence that the activity of the proteasome as well as of autophagy is altered early after TBI. While proteasome activity was significantly reduced in both hemispheres in CCI animals compared to sham conditions, the analysis of autophagy showed a rise in the activity in the ipsilateral hemisphere, while a decrease of activity could be observed in the contralateral hemisphere. Thus, the focal brain injury seemed to trigger changes in protein homeostasis in the ipsilateral hemisphere, which are comparable to observations made in ageing neural networks: Recently we showed that in ageing, a shift from proteasome activity towards autophagy can be observed and this mechanism was linked to differential modulation of chaperon Hsp-70 through BAG1/BAG3 [29,30,31]. Importantly, proteasome activity is crucial for synaptic plasticity both presynaptic and, prominently, postsynaptic [59,60,61,62] as it is involved in turnover and function of neurotransmitter receptors, scaffolding proteins in the post-synaptic density, post-synaptic signaling cascades [63] and protein translation [25]. Furthermore, impaired proteasome activity has been linked to neurodegenerative disorders such as Alzheimer’s disease [64] and impaired learning [65]. Recent research showed that particularly for l-LTP [66], proteasome activity, synergistically with brain-derived neurotrophic factor (BDNF), is necessary for consolidation [67,68]. Autophagy is known to contribute to changes in synaptic plasticity. While increased autophagy activation through mTOR inhibitors such as rapamycin [23,26,69] as well as quantum dots [27] and increased autophagy through microwave radiation [70] reduced LTP, also the reduction of autophagy might impair synaptic plasticity as it seems to be necessary for LTP [71,72]. Together, this suggests that a sensitive balance between protein degradation systems but also within individual protein degradation systems is required to maintain synaptic plasticity. To link these results to our electrophysiological data, we pharmacologically mimicked, in control animals, the protein degradation impairments observed after lesion. The activity assays demonstrated differential alterations of the two major protein degradation systems—while proteasome activity was impaired, autophagy was increased, being suggestive of a shift between the two degradation systems. We individually characterized these alterations by using pharmacological manipulation in control animals to mimic the alterations observed in TBI. Administration of a proteasome inhibitor or an autophagy activator lead to significantly impaired long-term synaptic plasticity in the somatosensory cortex, and this is in line with research on the hippocampus [23,73].

### 3.4. Applicability and Future Directions

Concluding, with rising incidence of traumatic brain injury as a global major cause of mortality and morbidity in human society, it is of great importance to better understand the early adaptive, cellular mechanisms of functional reorganization of the ipsi- and contralateral cortex during the acute phase of 24–48 h after TBI. In order to be able to develop improved treatment options, a better understanding of the pathophysiology of TBI is fundamental. In this regard, the present study provides new insights into the cellular mechanisms that could mediate later cognitive impairments after TBI on the cellular level. As we have shown here, the observed dysregulation of the proteasome degradation system early after TBI contributed to an impaired function of neural networks, specifically to cellular processes of learning and memory. Hence, our results could be applicable for further studies investigating potentially new therapeutical strategies during the acute phase after TBI by modulating the observed acute changes in protein homeostasis. With respect to the observed changes in long-term synaptic plasticity, one might consider in vivo electrophysiological experiments with combined optogenetic stimulation in a behavioral context in specific transgenic mouse lines to provide new insights into the cellular mechanisms of TBI-induced chronic impairments of cognitive functions.

## 4. Materials and Methods

### 4.1. Animals and Ethical Statement

All experiments of this study were performed in strict accordance with the European regulations and with the institutional guidelines of the Johannes Gutenberg University Mainz, Germany. The protocol was approved by the Animal Ethics Committee of the Landesuntersuchungsamt Rheinland-Pfalz (23 177-07/G 14-1-037). C57BL/6N wild-type mice of either sex at the age of 18–20 days (*n* = 131; electrophysiology: *n* = 42, molecular biology: *n* = 81, histology: *n* = 8) were housed at a constant room temperature of 23 ± 2 °C with a standard 12 h light/dark cycle and free access to food and water. The number of animals was kept to a minimum and all efforts were made to minimize the suffering of the mice.

### 4.2. Induction of Traumatic Brain Injury

Mice were initially anaesthetized with 4% isoflurane (AbbVie, Wiesbaden, Germany) and anesthesia was continuously maintained throughout the surgery by inhalation of 1.5% isoflurane mixed with oxygen. The mechanical brain trauma was induced as previously described in our lab by a controlled cortical impact (CCI) [11,74]. In brief, the mice were placed in a stereotactic frame before performing a 4 mm^2^ craniotomy over the right parietal sensorimotor cortex using a high-speed drill. A stereotaxic impactor for CCI (Impact One™, Leica Mikrosysteme, Wetzlar, Germany) was used to induce the mechanical trauma by positioning it perpendicular to the intact dura (impactor diameter: 1.5 mm, impact speed: 6 m/s, dwell time: 200 ms, impact depth: 0.8 mm). The previously removed bone flap was subsequently repositioned and sealed with histoacryl surgical glue (B. Braun-Melsungen, Melsungen, Germany) before sewing up the skin with polypropylene sutures (Ethicon, Somerville, MA, USA). The isoflurane flux was interrupted to terminate anesthesia and animals were placed back in their cage for at least 2 h recovery period under an infrared lamp. Sham-operated age-matched littermates were used as controls to compare the different experimental conditions.

### 4.3. Electrophysiology In Vitro

#### 4.3.1. Tissue Preparation

Animals were deeply anaesthetized with 4% isoflurane and decapitated at 1–2 days after lesion induction. The brains were quickly removed and placed in 4 °C artificial CerebroSpinal Fluid (aCSF) containing (in mM): 125 NaCl, 25 NaHCO_3_, 2.5 KCl, 1.5 MgCl_2_, 2 CaCl_2_, 1.25 NaH_2_PO_4_, and 25 D-glucose, equilibrated with 95% O_2_ and 5% CO_2_ (pH = 7.4). Acute coronal slices with a thickness of 300 µm were prepared from the somatosensory cortex using a vibratome (Leica VT-1000-S, Leica Mikrosysteme, Wetzlar, Germany) and incubated at room temperature for 15 min in oxygenated aCSF.

#### 4.3.2. Multi-Electrode Array Recordings

Neuronal synaptic activity in the acute slices was recorded in both cortical hemispheres by use of perforated Multi-Electrode Array chips (pMEA) (pMEA32S12 Layout 4, Multichannel Systems, Reutlingen, Germany) equipped with 32 recording electrodes (4 rows of 8 electrodes) and 12 stimulating electrodes (2 rows of 6 electrodes). The acute slices were transferred into a circular perfusion chamber mounted on the pMEA chip and incubated for 90 min at 35 °C in oxygenated aCSF. Slices were carefully positioned in order to put the recording electrodes below the supra-granular cortical layers 2/3 and one row of stimulation electrodes in layer 4. To secure the position of the slice in the recording chamber and improve contact between slices and electrodes, a low negative pressure (4–7 mbar) was constantly applied through the perforation using a constant vacuum pump (Multi channel Systems, Reutlingen, Germany). The center of the pMEA chip was placed at around 1 mm distance from the border of the lesion and in homotopic regions when recorded in the contralateral hemisphere of injured animals or in sham-operated mice. Each pMEA chip was used for multiple recordings of several brain slices including measurements of CCI-treated animals and of the respective sham control.

All electrophysiological signals in the slices were acquired at 35 °C under constant perfusion of oxygenated aCSF. The extracellularly recorded synaptic signals (field potentials, FP) were evoked by stimulating afferent fibers in layer 4 using the integrated electrical stimulus generator of the MEA2100-headstage (Multi channel Systems, Reutlingen, Germany). The stimulation pattern was designed and controlled through MC_Rack and MC_Stimulus (Multichannel System, Reutlingen, Germany) as a biphasic voltage signal (100 μs/phase). Baseline extracellular FPs were recorded every 60 s through a paired-pulse protocol (interpulse interval: 20 ms) for at least 30 min before long-term potentiation (LTP) induction and were similar in all experimental groups. LTP was induced in slices by adapting a theta-burst stimulation (TBS) protocol previously used in our lab [47]. The TBS protocol consisted of three trains (at 30 s intervals) of five bursts (at 5 Hz) each providing four stimuli at 100 Hz. In order to induce l-LTP, this TBS protocol was repeated four times with an intermission of 5 min. The lesion-induced changes in long-term synaptic plasticity were assessed by applying paired-pulse stimulations at 90 s intervals post-TBS. Raw signals were acquired, at a sampling rate of 50 kHz, with a MEA2100-acquisition system. To optimize detection, the signal was band-pass filtered between 5 and 2500 Hz. Offline analyses were performed with the software MC_Rack and pCLAMP 10 software (Molecular Devices, Sunnyvale, CA, USA).

Pharmacological inhibition of the proteasome was induced by adding the proteasomal inhibitor MG132 (Cayman Chemical, Ann Arbor, MI, USA) at a final concentration of 10 µM to the aCSF. Cortical slices were incubated with MG132 for 60 min prior to the LTP-induction protocol (TBS), and afterwards the bathing solution was switched back to normal aCSF. In order to activate autophagy in the cortical slices, the tissue was perfused with aCSF containing rapamycin (Enzo, Farmingdale, NY, USA) at a final concentration of 1 µM for 60 min pre-TBS and 30 min post-TBS. The bathing solution was then switched back to aCSF for the rest of the recording period.

### 4.4. Histology

Some brain slices (*n* = 4) were immediately fixated after the preparation by use of 4% paraformaldehyde (Carl Roth, Karlsruhe, Germany) for 2 h. Cryoprotection was operated overnight in 30% sucrose in phosphate-buffered saline (PBS) and the fixed brain slices were sectioned at 50 μm using a microtome (Leica CM1325, Leica Mikrosysteme, Wetzlar, Germany). Brain sections were Nissl-stained and subsequently mounted in Entellan^®^ New (Merck Millipore, Billerica, MA, USA) before imaging with a stereo microscope M205 FA (Leica Mikrosysteme, Wetzlar, Germany).

### 4.5. Western Blots

Protein quantification was performed with lysates from cortical probes prepared from regions homotopic to the electrophysiological recordings from 12 mice for each condition (*n* = 36). Proteins were electroblotted using 12% sodium dodecyl sulfate polyacrylamide gel electrophoresis (SDS-PAGE) gel to a polyvinylidene difluoride (PVDF) membrane. The primary antibodies, CaMKII (C6974, Sigma Aldrich, Saint-Louis, MO, USA), pCaMKII (#3361, Cell Signaling Technologies Inc., Danvers, MA, USA) and GAPDH (A300-641A, Bethyl laboratories, Montgomery, AL, USA), were incubated overnight at 4 °C. For detection, secondary horseradish peroxidase(HRP)-conjugated anti-rabbit (A120-201C2, Dianova, Hamburg, Germany) was used and imaged using ECL detection solution (Merck Millipore, Darmstadt Germany) and acquired with Image Lab 2.0 software (Bio-rad, Hercules, CA, USA). Full uncut blots are provided in Figure S1.

### 4.6. Enzymatic Assays

#### 4.6.1. Cell Death (TUNEL) Assay

After deep anesthesia with 4 % isoflurane, animals (*n* = 4) were sacrificed and the removed fresh brains were flash frozen in pre-cooled 2-Methylbutane (Sigma-Aldrich, Saint-Louis, MO, USA). Frozen brains were subsequently embedded in CryoGlue medium (SLEE medical, Mainz, Germany) and coronal slices of the somatosensory cortex were performed (20 μm thick) using a freezing cryostat (SLEE medical, Mainz, Germany). The In Situ Cell Death Detection Kit, TMR Red (Roche, Basel, Switzerland) and the protocol from the supplier were used for the detection of cell death through the labeling of DNA strand breaks. After staining, the slices were mounted in Immunoselect Antifading Mounting Medium DAPI (SCR-38448, Dianova, Hamburg, Germany) and imaged with a confocal microscope (Leica TCS SP5, Leica Mikrosysteme, Wetzlar, Germany).

#### 4.6.2. Proteasome Activity Assay

Acute cortical slices (300 μm) from sham-operated and lesion-induced animals were prepared as previously described and the cortical tissue of interest immediately dissected (at 1 mm from the border of the lesion and in the homotopic region in contralateral hemisphere and sham-operated mice). The tissue was ruptured in a lysis buffer containing (in mM): 10 4-(2-hydroxyethyl)-1-piperazineethanesulfonic acid (HEPES), 10 potassium acetate, 1.45 magnesium acetate and 1 dithiothreitol (DTT) (pH = 7.6). Samples were then incubated at 4 °C for 30 min and centrifuged at 5000 rpm for 5 min at 4 °C. After the determination of total protein concentration, 10 μg of proteins were mixed in the assay buffer containing (in mM): 15 HEPES, 130 potassium acetate, 1.5 magnesium acetate, 1.5 CaCl, 2 DTT, 8 adenosine triphosphate (ATP), 0.1 Suc-LLVY-AMC (Enzo Life Sciences, Farmingdale, NY, USA). The reaction mix was then transferred to a 96 Well Polystyrene Black Microplate (Greiner Bio-One, Kremsmünster, Austria) and placed in the Wallac 1420 VICTOR2 Microplate Reader (Perkin Elmer, Waltham, MA, USA). After 2 h of incubation at 37 °C, the connected software enabled to measure the 7-amino-4-methylcoumarin (AMC)-mediated fluorescence at 360 nm, as previously described in our group [75]. For data analysis, the autofluorescence of the fluorogenic substrate was subtracted and the proteasome activities values of lesion samples were normalized to the one of sham: $Ratio=\frac{F\;\left(\mathrm{Lesion}\right)\;\times \;100}{F\;\left(\mathrm{Sham}\right)}$. 

#### 4.6.3. Autophagic Degradation Activity Assay

Acute cortical slices (300 μm) from sham operated and lesion-induced animals were prepared as previously described and directly incubated for 2 h at room temperature either in aCSF or in aCSF supplemented with 200 μM chloroquine, both equilibrated with 95% O_2_ and 5% CO_2_. After incubation, the ipsi- and contralateral hemispheres were separated and flash-frozen in liquid nitrogen. Western blot analysis was carried out as described previously [75]. Analysis was performed with the Fusion-SL 3500 WL system (Peqlab) and Aida Image Analyzer v.4v26 software (raytest). The following antibodies were used: LC3B (L7543, Sigma-Aldrich) and Tubulin (T9026, Sigma-Aldrich).

### 4.7. Statistical Analysis

Results are presented as mean ± SEM. The statistical evaluation of the data was performed with GraphPad Prism 7 (GraphPad Software, San Diego, CA, USA). To test for normal distribution of the data, the Kolmogorov–Smirnov test in association with the Shapiro–Wilk test were performed. If the data were normally distributed, one-way ANOVA and post hoc least significant difference (LSD) were used to compare sham, lesion ipsilateral and lesion contralateral. In case data were not normally distributed, a one-way Kruskal–Wallis test and pairwise Mann–Whitney U test were employed. Significantly different values are indicated by asterisks in the figures (*: *p* < 0.05; **: *p* ≤ 0.01; ***: *p* ≤ 0.001).

### 4.8. Limitations

This study used an established ex vivo-in vitro model for traumatic brain injury, which is clinically relevant [76]. However, one should keep in mind that the lissencephalic structure and the relatively young age of the mice in the present study limits a direct applicability to the manifold variety of traumatic brain injuries in humans. The role of TBI on long-term synaptic plasticity at laterstages post-injury could not be investigated, since the electrophysiological results plus the induced strength of l-LTP were different in older control mice. Furthermore, we observed a reduced survival rate in more aged animals post TBI.

## Supplementary Materials

Supplementary Materials can be found at https://www.mdpi.com/1422-0067/20/12/3048/s1.

## Abbreviations

aCSF	artificial cerebrospinal fluid
AMC	7-amino-4-methylcoumarin
ATP	Adenosine triphosphate
BAG	Beclin2-associated anthanogene
BDNF	brain-derived neurotrophic factor
CaCl_2_	calcium chloride
CaMKII	calcium/calmodulin-dependent protein kinase II
CCI	controlled cortical impact
CO_2_	carbon dioxide
DNA	deoxyribonucleic acid
DTT	dithiothreitol
EFP	extracellular field potentials
GABA	gamma-aminobutyric acid
HEPES	4-(2-hydroxyethyl)-1-piperazineethanesulfonic acid
Hsp	heat shock protein
KCl	potassium chloride
LC3	light chain 3 protein
LFP	local field potential
L-LTP	late long-term plasticity
LTP	long-term plasticity
MEA	multi electrode array
MgCl_2_	magnesium chloride
mTOR	mammalian target of rapamycin
n	number
N_2_	nitrogen
NaCl	sodium chloride
NaH_2_PO_4_	monosodium phosphate
NaHCO_3_	sodium hydrogene carbonate
NMDA	N-methyl-d-aspartic acid
O_2_	oxygen
pMEA	perforated multi-electrode array
TBI	traumatic brain injury
TBS	theta-burst stimulation
