# Supplementary material for: Proteasome and Autophagy-Mediated Impairment of Late Long-Term Potentiation (l-LTP) after Traumatic Brain Injury in the Somatosensory Cortex of Mice
Source: Int J Mol Sci. 2019 Jun 21;20(12):3048. doi: 10.3390/ijms20123048 (PMC6627835; doi:10.3390/ijms20123048)
Supplement: Supplementary file 1 [file ijms-20-03048-s001.pdf]

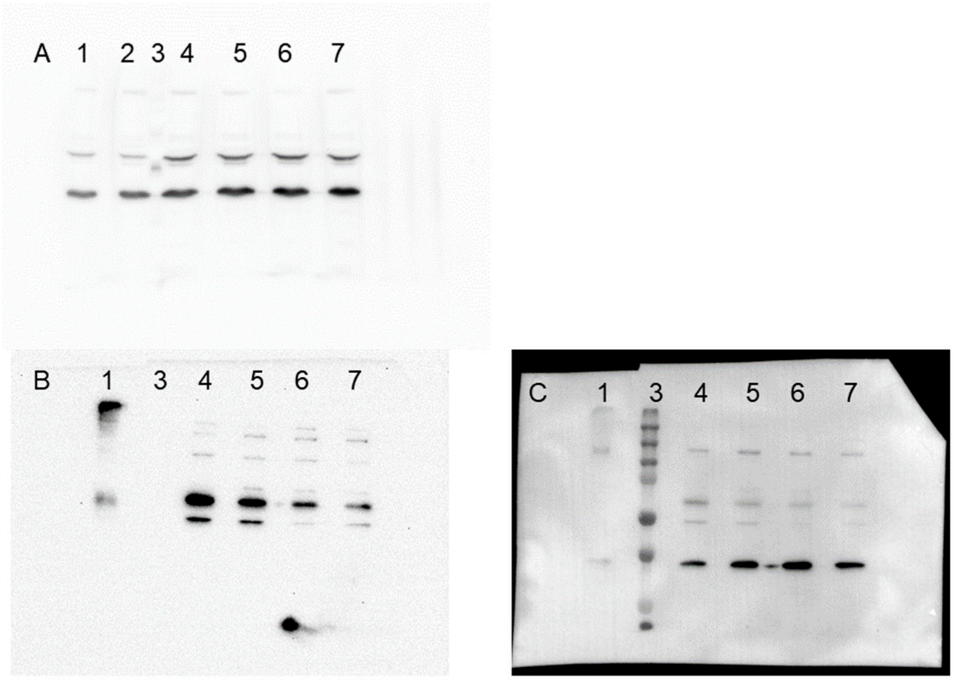


**Figure S1. Full uncut Western blots for plasticity related proteins.** Full uncut blots are presented for the representative Western blots featured in the present study. The numbers on top of each column correspond to the following probes: 1 = control brain lysate, 2 = control brain lysate, 3 = marker, 4 = lesion ipsilateral, 5 = lesion contralateral, 6 = sham, 7 = sham. Control brain lysates were not included in the analysis but used to assess the quality of the Western blots. The mean of the two sham values was used to normalize the different experimental conditions. **(A)** Representative immunoblot for CaMKII showing different bands, most prominently CaMKII$\alpha$ at approximately 54 kDa and the housekeeping gene GAPDH at 36 kDa. **(B)** and **(C)** show exemplary Western blots for pCaMKII. Since the band intensities were very different, different imaging times were used for the phosphorylated antibody (B) and GAPDH (C) in order to avoid oversaturation and then always normalized to the individual blot's housekeeping genes.
